# Draft Genome Sequence of the Polychlorinated Biphenyl Degrader Comamonas testosteroni Strain YAZ2, Isolated from a Natural Landscape in the Tohoku Region of Japan

**DOI:** 10.1128/mra.00806-21

**Published:** 2022-01-13

**Authors:** Tomijiro Hara, Yumiko Takatsuka, Yuh Shiwa, Kenji Yokota

**Affiliations:** a Environmental Microbiology Research Section, Laboratory for Complex Energy Processes, Institute of Advanced Energy, Kyoto University, Gokasho, Uji, Kyoto, Japan; b Department of Molecular Microbiology, Tokyo University of Agriculture, Setagaya, Tokyo, Japan; c Department of Agricultural Chemistry, Tokyo University of Agriculture, Setagaya, Tokyo, Japan; University of Southern California

## Abstract

We report a draft genome sequence of Comamonas testosteroni strain YAZ2, a polychlorinated biphenyl (PCB) degrader that was isolated from a PCB-unpolluted environment. The assembled genome contains a single 5.4-Mb chromosome and an 87-kb plasmid. The *bph* gene cluster, which is involved in PCB degradation, was found on the chromosome.

## ANNOUNCEMENT

Comamonas testosteroni strain YAZ2 is a polychlorinated biphenyl (PCB) degrader that was isolated from PCB-uncontaminated soil, collected in Yonezawa (37°54′3.76″N, 140°6′13.01″E) in the Tohoku region of Japan, by enrichment culture using biphenyl as the sole carbon source (1). W-minimal medium was used as the isolation medium, as described by Kimbara et al. (2). This strain was shown to degrade 5 mg/L PCBs with 50% 3-chlorine substitution by 83.1 ± 1.5% (1).

Genomic DNA was extracted from YAZ2 cells freshly cultured in W-medium supplied with biphenyl using a bacterial DNA preparation-solution kit (Jena Bioscience, Jena, Germany). The extracted DNA was confirmed to be of sufficient quality for next-generation sequencing (NGS) using a Thermo Fisher Scientific NanoDrop spectrophotometer (purity), Invitrogen Qubit fluorometer (double-stranded DNA [dsDNA]), and Agilent 2200 TapeStation system (electrophoresis) (Hokkaido System Science Co., Ltd., Hokkaido, Japan). The genome was sequenced using (i) an Illumina HiSeq platform with 350-bp paired-end and 6- to 10-kb mate-pair libraries (Hokkaido System Science Co., Ltd.) and (ii) a Roche 454 GS Junior sequencer (Roche Diagnostics K.K., Tokyo, Japan). The Illumina paired-end and mate-pair libraries yielded 21,847,630 and 10,944,900 paired-end 100-bp reads, respectively, and the Roche 454 library yielded 202,587 reads, with an average read length of 444 bp. Sequence assembly of the YAZ2 genome was performed cooperatively by Bits Co., Ltd. (Tokyo, Japan). Default parameters were used for all software unless otherwise specified. The raw reads from Illumina sequencing were trimmed using Cutadapt v1.1 (3) and Trimmomatic v0.32 (4) and subsequently assembled using Velvet v1.2.10 (5), resulting in 55 contigs/scaffolds, amounting overall to 5,661,919 bp, with an *N*_50_ value of 4,645,585 bp. The remaining gaps were filled by manual recursive mapping of Illumina paired-end reads against the contigs/scaffolds (minimum number of reads, 10) (6). Further scaffolding by manual mapping of Illumina mate-pair reads linked 3 of the 55 contigs/scaffolds into 1 scaffold, and the remaining unlinked contigs were removed from the assembly. Because the resulting assembly did not contain any plasmid sequences, Roche 454 sequence reads were assembled using the NewblerGS *de novo* assembler v3.0, yielding 118 contigs. Among these contigs, 1 contig (87 kb), with the start and end overlapping with 1 Illumina contig, showed the highest similarity, based on BLASTN analysis, to the plasmid sequence of *Acidovorax* sp. strain JS42 (GenBank accession number NC_008765), belonging to the same family as C. testosteroni, i.e., *Comamonadaceae*. Thus, this contig was determined to be a plasmid sequence. Annotation analysis was performed to identify the proteins and estimate their functions using the DFAST v1.4.0 pipeline with default settings, excluding the Prodigal and tRNAscan-SE options (https://dfast.ddbj.nig.ac.jp) (7).

Overall, the draft genome sequence of YAZ2 contained a single chromosome in one scaffold (5,448,456 bp, with a gap ratio of 0.38%, a G+C content of 61.6%, and coverage of 607.9×) generated from the Illumina data and one plasmid sequence (87,186 bp, with a G+C content of 62.4% and coverage of 1,032.5×) generated from Roche 454 sequence data. It also contained 5,001 putative protein-encoding genes, 4 rRNAs, and 79 tRNAs; the *bph* operon (*bphEGFA1A2A3BCDA4*), the gene cluster involved in PCB degradation, was found on the chromosome.

### Data availability.

This whole-genome shotgun project has been deposited in DDBJ/ENA/GenBank under the accession numbers AP024703 (chromosome) and AP024827 (plasmid). The raw sequence data have been deposited under accession numbers DRA012593 and DRA012754.
